# Data Acquisition, Analysis and Transmission Platform for a Pay-As-You-Drive System

**DOI:** 10.3390/s100605395

**Published:** 2010-06-01

**Authors:** Luciano Boquete, José Manuel Rodríguez-Ascariz, Rafael Barea, Joaquín Cantos, Juan Manuel Miguel-Jiménez, Sergio Ortega

**Affiliations:** Department of Electronics, University of Alcalá, 28871 Alcalá de Henares, Madrid, Spain; E-Mails: jmra@depeca.uah.es (J.M.R.-A.); barea@depeca.uah.es (R.B.); jjcantos@gmail.com (J.C.); jmanuel@depeca.uah.es (J.M.M.-J.); sergioortegarecuero@hotmail.com (S.O.)

**Keywords:** Pay As You Drive, EOBD, GPS, UMTS, RF Harvesting

## Abstract

This paper presents a platform used to acquire, analyse and transmit data from a vehicle to a Control Centre as part of a Pay-As-You-Drive system. The aim is to monitor vehicle usage (*how much*, *when*, *where* and *how*) and, based on this information, assess the associated risk and set an appropriate insurance premium. To determine vehicle usage, the system analyses the driver’s respect for speed limits, driving style (aggressive or non-aggressive), mobile telephone use and the number of vehicle passengers. An electronic system on board the vehicle acquires these data, processes them and transmits them by mobile telephone (GPRS/UMTS) to a Control Centre, at which the insurance company assesses the risk associated with vehicles monitored by the system. The system provides insurance companies and their customers with an enhanced service and could potentially increase responsible driving habits and reduce the number of road accidents.

## 1. Introduction

This paper presents an on-board vehicle platform used to acquire, analyse and transmit data to a Control Centre (CC) as part of a Pay-As-You-Drive (PAYD) system. Advances in communications technology and development of low-cost, high-performance hardware and location systems enable implementation of a diverse range of driving applications. For example, Lin *et al.* presenteo d a system used to monitor vehicle exhaust emissions, in which data acquired by the vehicle’s European On-Board Diagnostics (EOBD) system are passed through an RS-232 converter, processed by a microcontroller and then transmitted to a server over a General Packet Radio Service (GPRS) cellular network [1]. Sa *et al.* described a system that uses a microcontroller connected to a laptop computer to acquire data about vehicle speed, acceleration and fuel consumption, among other parameters [2]. Project AIDER presented a portable system based on sensors and video cameras that, in the case of an accident, delivers data on the incident’s circumstances and severity (as well as on the occupants’ state of health) to the appropriate authorities via a cellular network (GPRS) [3]. The UTDrive project’s aim is to understand driving behaviour (driver identification, driver distraction detection and driver manoeuvre recognition) using multi-channel sensor data from CAN-bus, Global Positioning System (GPS), video, audio and additional accelerator- and brake-pedal-pressure signals [4]. Moreover, many contemporary vehicles include driver assistance systems, such as lane departure warning (LDW) mechanisms, pedestrian detection, traffic sign detection (TSD), *etc.*

The aim of this paper is to implement a system comprising interfaces (EOBD, GPS, GPRS, Universal Mobile Telecommunications System– UMTS, *etc.*) and computing capacity required to acquire, process and transmit data to a CC as part of a PAYD system. The architecture proposed could also be adapted to work in conjunction with other services. PAYD services require access to vehicle usage data (*how much*, *when*, *where* and *how*). These are acquired in the vehicle and then transmitted via a communications system based on mobile telephone technology. This paper’s principal contribution is that the system implemented also provides information about driving style (*how* a vehicle is driven) and analyses habits directly related to the level of risk assumed by the driver.

## 2. Pay As You Drive

This section describes PAYD vehicle insurance and its potential impact on vehicle usage and safety. Under the PAYD model, the cost of automobile insurance is determined by vehicle usage. The simplest form of PAYD bases the insurance cost on the number of kilometres driven during the policy term, thereby creating the option for customers to lower the premium by reducing the distance travelled. PAYD insurance can also provide savings for responsible customers, reflecting the idea that price should reflect costs [5–9]. It has been suggested that PAYD systems could reduce vehicle travel by over 10% [6] thereby also reducing the road accidents, traffic congestion, road and parking infrastructure costs, energy consumption, pollutant emissions and consumer costs associated with vehicle use.

The PAYD insurance model may also take other data into account in addition to number of kilometres travelled, such as *when, where* and *how* the vehicle is driven. For example, it may be suggested that vehicle usage is likely to involve greater risk at night and at times of the day or year when traffic is heaviest. The geographic location of habitual vehicle usage may also affect the level of risk assumed. Another factor likely to influence the risk level is *how* the vehicle is driven, as driving style and habits, which have received little attention to date, have a significant influence on accident rates. Several studies show that human factors are responsible for 90% of road accidents and that 30% of these are caused by distractions (using a mobile telephone, changing radio station, putting out a cigarette, *etc.*) [10,11].

Speeding is one of the principal human factors affecting driving risk and various studies show the advantages of using speed management systems to improve road safety. In the White Paper “European Transport Policy for 2010: Time to Decide”, the European Commission draws attention to the fact that road transport is the most dangerous and costly mode in terms of loss of human life (“one person in three will be injured in an accident at some point in their lives”) [12]. If vehicle users are aware that speeding will incur economic penalties enforced via their insurance premiums, they may be more likely to decrease their speed, thereby contributing to reducing the possible effects of an accident [13].

The disadvantage of this model is that it only assesses codified driving risk, not actual driving risk. For example, a driver who regularly breaks the speed limit would be more heavily penalized than one who regularly drives within it. However, this does not consider context and a driver who regularly breaks the speed limit but otherwise drives in a safe manner would be charged less than a customer who regularly drives within the speed limit but drives erratically or carelessly. It may also be suggested that if this type of system were widely installed in vehicles, drivers would feel more secure, as they would deduce that the penalties applied would be likely to make other road users drive more responsibly.

The aim of this paper is to develop a system that identifies how much, when, where and how the user drives the vehicle. This information can be easily obtained from data acquired from the CAN Bus (EOBD), the GPS and a circuit designed to detect driver mobile-telephone use. Information indicating ‘how much’ (time, number of kilometres) is obtained from a microcontroller, from the GPS and from the EOBD. The GPS also provides information on ‘when’ and ‘where’ the vehicle is used. Access to information captured by the EOBD enables the number of vehicle occupants to be estimated. This data is of interest because a higher number of vehicle occupants indicates a higher cost for the insurance company in the case of an accident.

Determining ‘how’ the vehicle is used is an open research topic in which many factors have an influence (driver state and experience, vehicle type, driving style, *etc.*), although this research paper has addressed this issue by analysing the following parameters:

- Respect for speed limits: GPS data and current speed are checked against the road database to ascertain the current speed limit and then check if the vehicle is within it.
- Driving style: This paper suggests accelerating and braking hard demonstrates an aggressive driving style. The system analyses these two factors to judge if the vehicle is being driven aggressively. It also estimates the number of occupants per trip by detecting the number of doors opened and seatbelts fastened.
- Driver mobile-telephone use: Many studies show that driver mobile-telephone use increases driving risk [14–17]. This risk also extends to pedestrians [18]. It is estimated that mobile telephone use for one hour per month increases the risk of having an accident by 400–900% [19]. A Dutch study estimates that zero mobile-telephone use whilst driving would prevent nearly 600 road deaths and hospital admissions per year in the Netherlands (2004 data), while a US study estimates that telephone use whilst driving in the US results in around 2,600 deaths and 330,000 serious injuries per year [20].

## 3. System Architecture

**Figure 1.**
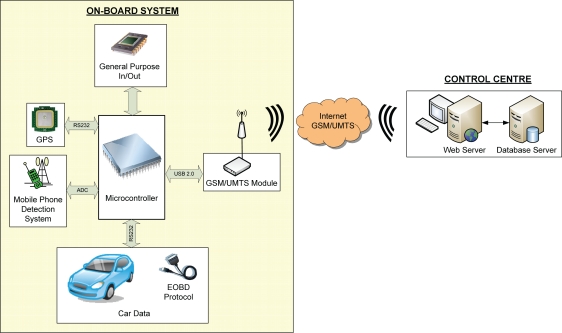
Block diagram of the system.

This section presents an overview of the implemented system (Figure 1), which comprises an On-board System (OS) installed in a vehicle and a CC. The system uses mobile telephony to transmit data between the OS and the CC. The OS acquires vehicle function data from the EOBD system, vehicle position-speed data from the GPS and driver mobile-telephone use data from a detector circuit (RF energy scavenging). The core of the OS is a high-performance microcontroller that processes and stores the data captured before transmitting it to the CC.

**Figure 2.**
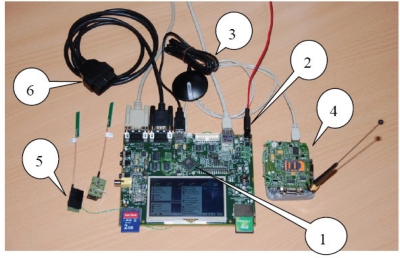
Development platform.

Figure 2 shows an image of the platform implemented, which comprises the following components:

1. Processor: AT91SAM9M10 (400-MHz ARM926 core, 128 MB of SDRAM and 4 GB of NAND Flash memory) with a series of integrated peripherals (USB, Ethernet, SD/MMC card reader, serial interfaces, *etc.*). The system also includes a 3.5” touch-screen (only used during development and debugging). The platform uses the Linux operating system to implement the required functions (GPS data processing, mobile telephone communications, signal processing, data file management, *etc.*).
2. Power supply: Dual source (vehicle and device battery) to ensure device operation if the car battery is disconnected.
3. GPS module: Commercially available model (EM408, based on the SiRF Star III chipset) connected to the microcontroller via a Universal Asynchronous Receiver/Transmitter (UART) interface and configured as a 4800-bps, 8-bit, non-parity serial line. The NMEA 0813 standard is used to obtain the data. This standard allows for code portability and replacement of the GPS module without having to modify the rest of the platform.
4. MTX-H2 modem: The UMTS and High Speed Downlink Packet Access (HSDPA) protocols are used to transmit and receive data between the OS and the CC. This module is controlled by the microcontroller using AT commands (Attention command and standardized modem control commands).
5. RF harvesting block (mobile-telephone detection circuit): Electronic system used to detect driver mobile-telephone use.
6. EOBD connection: Achieved via a bespoke interface based on the ELM327 integrated circuit and capable of communicating via any protocol currently in use (*i.e.*, ISO 9141-2, ISO 14230-4, SAE J1850 (PWM and VPM), ISO 15765-4 and SAE J1939). Data is converted into packets transmitted to the microcontroller via an RS-232 serial interface. This enables the platform to acquire the data needed from the EOBD system, which monitors the vehicle’s various parameters via a series of sensors and generates and logs warnings in case of malfunction. Examples of the data available include engine revolutions per minute (RPM), fuel consumption, number of seatbelts fastened, *etc.*
7. Control Centre. The CC consists of two servers, a web server (Apache Tomcat server), which hosts the application used to communicate between the vehicles and the graphic interface, and a database server (MySQL database server), which stores and processes the data used to assess each vehicle’s level of risk [21,22]. The CC can be configured to inform users by e-mail or Short Message Service (SMS) about the estimated level of risk over a set time period. This feedback could increase user awareness about the risk associated with certain driving habits and encourage improvement.

**Figure 3.**
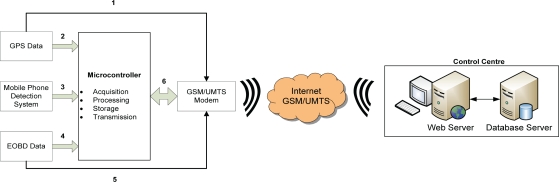
Information flow.

Figure 3 shows the information flow designed to exploit the hardware architecture’s potential and the microcontroller’s processing capacity. Under normal driving conditions, the microcontroller acquires data from the GPS (speed and geographic position) and checks whether the driver is respecting the speed limit (path 2). At the same time, it checks whether the driver is using a mobile telephone (path 3) and also acquires data from the vehicle’s EOBD system (RPM, number of doors opened and number of seatbelts fastened), from which it estimates the number of passengers (path 4). This data is processed and stored in the microcontroller prior to transmission to the CC for analysis.

Provided a mobile telephone signal is available, the CC can check the vehicle’s real-time position (path 1) to provide additional services such as fleet monitoring, vehicle location in case of theft, *etc.* Furthermore, users at the CC can access the EOBD protocol (path 5) to obtain vehicle data for diagnostic and preventive maintenance purposes. This data could also be used to provide new services in the future, such as remote tachometry.

## 4. Detection of Driver Mobile-Telephone Use

**Figure 4.**
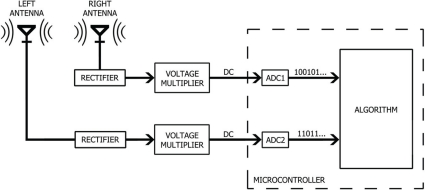
Diagram of the RF harvesting blocks.

Detecting mobile telephone use within a defined physical space requires an energy-capturing circuit tuned to an appropriate frequency. This process is known as RF harvesting or RF energy scavenging. In its simplest form, this circuit is known as a “rectenna”, *i.e.*, a combined antenna and rectifier producing DC power from RF-electromagnetic energy [23]. The antenna tunes in to the selected frequency bands (Global System for Mobile Communications (GSM), UMTS) and the voltage is amplified by a voltage multiplier and then rectified [24,25]. Low-threshold RF Schottky diodes are used to maximize the rectifier’s voltage output. The rectified DC voltage is stored in a large capacitor and digitized by an analogue-to-digital converter for subsequent storage and processing by a microcontroller (Figure 4).

**Figure 5.**
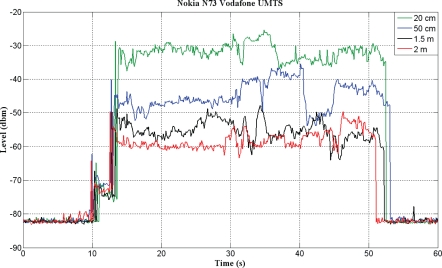
Energy captured over various open-air distances.

The voltage obtained with this system depends, among other factors, on the distance between the telephone and the antenna and the relative orientation between the two. Figure 5 shows a practical example of the energy captured from a mobile telephone over various open-air distances while a call is being made. When the telephone is not in use, the energy captured is minimal. When a call is made, this energy is inversely proportional to the distance between the telephone and the energy-capturing antenna. This voltage can be digitized by a microcontroller to enable subsequent application of a detection algorithm to estimate mobile telephone use.

**Figure 6.**
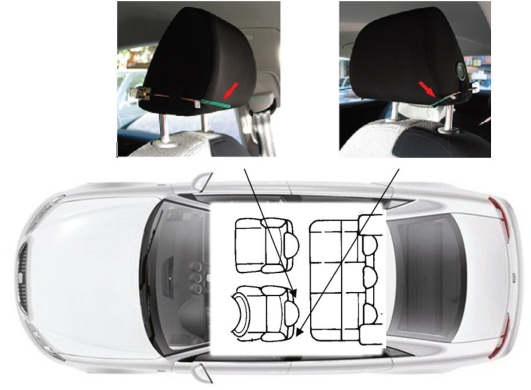
Antennae location within the vehicle.

The vehicle’s internal structure and metal chassis interfere with the theoretically ideal distribution of the mobile system’s energy (energy proportional to telephone proximity). Therefore, a 2-antenna system (right and left) is used and each antenna has its own energy-capturing circuit (Figure 6). When positioned suitably within the vehicle, this set-up, combined with appropriate analysis of the signals captured, reliably detects driver mobile-telephone use.

**Figure 7.**
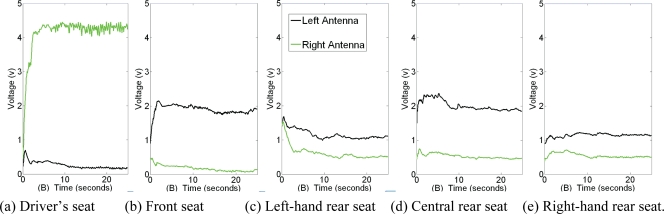
Energy captured by the rectenna circuit.

The system was tested in a car driven on the University of Alcalá campus. A Sony-Ericsson K800i mobile telephone subscribed to Spanish operator Movistar was used. Figure 7 shows system performance when a single mobile telephone was used inside the vehicle by (a) the driver, (b) the front-seat passenger, (c) the left-hand rear-seat passenger, (d) the central rear-seat passenger and finally (e) the right-hand rear-seat passenger.

As can be seen, the strongest signal was captured when the telephone was used by the vehicle driver (>4 volts in the antenna closest to the mobile telephone). When the telephone was used by other vehicle occupants, the voltage levels captured were usually below 2 volts. This difference therefore enables the system to detect when the telephone is used by the vehicle driver. The mobile telephone usage detection algorithm is executed permanently in the microcontroller, allowing the system to monitor the number, time and duration of all calls made whilst the vehicle is in motion. Driver mobile-telephone usage time (T_MOBILE_) is then used as a risk assessment criterion.

## 5. Non-Aggressive *Versus* Aggressive Driving

Other factors used to assess vehicle usage are respect for speed limits and driving style (aggressive and non-aggressive). Below are the results of real-world tests performed by drivers purposely adopting aggressive and non-aggressive driving styles. The route selected included various elements that required changes in driving speed and featured several potential distractions (roundabouts, pedestrian crossings, *etc.*). The tests took place on the University of Alcalá campus over a 2.6 km route. The speed limit for the entire route was 50 km/h and the test circuit was closed to all other traffic and pedestrians.

**Figure 8.**
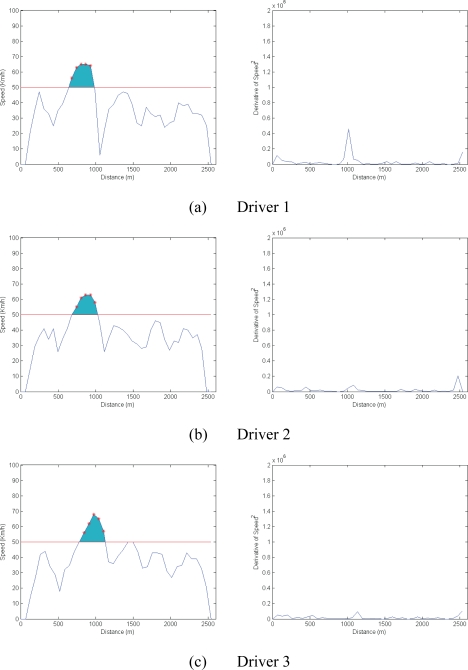
Results of non-aggressive driving.

The test assessed the vehicle speed parameter, which was obtained from the GPS. Figure 8 shows the speed graphs for the first three drivers. These reveal that these drivers exceeded the speed limit for brief periods (shown in red) and drove non-aggressively (shown by the square of the derivative of speed, also in Figure 8).

**Figure 9.**
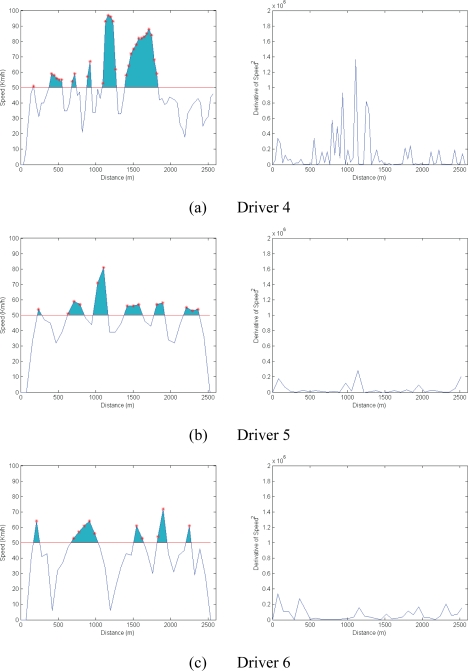
Results of aggressive driving.

In contrast, Figure 9 shows the results of more pronounced speeding and an aggressive driving style characterized by erratic accelerating and braking (again shown by the square of the derivative of speed). To analyse these aspects mathematically, the S_SPEED_ and S_ACCELERATION_ variables have been defined. S_SPEED_ is the area comprising the difference between the horizontal line indicating the speed limit (50 km/h in this example) and the extent by which this threshold is exceeded (shown in blue in Figures 8 and 9). S_ACCELERATION_ is the integral of the square of the derivative of speed.

In general terms, aggressive drivers drive erratically and at excessive speed, change gear at high RPM and have to brake sharply if they encounter unanticipated situations, such as an inattentive pedestrian on a pedestrian crossing. In contrast, non-aggressive drivers drive moderately and at permitted speeds, enabling them to respond better to unanticipated hazards encountered on the road.

Table 1 quantifies driving style numerically. Drivers 1, 2 and 3 exceed the speed limit at certain points on the route and their driving erraticism is measured as being 8.45 × 10^4^, 5.21 × 10^4^ and 4.64 × 10^4^ km/h^2^. For drivers 4, 5 and 6, the figures are clearly higher (3.11 × 10^5^, 1.00 × 10^5^, 1.74 × 10^5^), suggesting they take greater risks when driving.

## 6. Data Processing

The aim is to acquire statistical data on vehicle usage that can be used by insurance companies to set insurance premiums on the basis of the risk assumed by each individual customer. Although the type of data considered and analysis of the same are beyond the scope of this paper, Table 2 suggests some possible criteria.

Based on the data in Table 2, a possible risk assessment calculation is shown below:

$$\begin{array}{l} \mathrm{Cost}=C_{F}+k_{1}.N_{K}.\;\frac{\left(k_{2}.\%\;T_{Z1}+k_{3}.\%\;T_{Z2}\right)}{100}.\frac{\left(k_{4}.\%T_{\mathrm{DAY}}+k_{5}.\%T_{\mathrm{NIGHT}}\right)}{100}.[1+k_{6}.(N_{\mathrm{OCCUPANTS}}-1)]+\\ +\;k_{7}.T_{\mathrm{MOBILE}}+k_{8}.A_{\mathrm{SPEED}}+k_{9}.S_{\text{ACCELERATION}}; \end{array}$$

where:

*C_F_*: Fixed charge.
*N_K_*: Number of kilometres travelled by the vehicle.
*%T_Zi_*: Percentage of time the vehicle is in zone i (based on a two-zone division).
*%T_DAY_, T_NIGHT_*: Percentage of time the vehicle is used during the day/night.
*N_OCCUPANTS_*: Average number of vehicle occupants.
*k_1_,…k_9_*: Appropriate constants.

To make the insurance cost proportionate to risk and usage, the premium’s variable charges could be based on criteria such as number of kilometres travelled, zone, day/night usage, number of occupants, mobile telephone use, driving style and respect for speed limits.

By way of example, Table 3 shows usage data for two vehicles (A and B) over a one-month period (30 days). Vehicle A is mainly used to travel between home and work in the same city during the day. Vehicle B is used by a sales representative who travels between several provinces (sometimes at night) and regularly exceeds the speed limit, drives aggressively and uses a mobile telephone whilst driving. Based on the suggested formula, vehicle A’s insurance premium would be €47.20, while vehicle B’s would be €130.67. This calculation may be modified according to commercial strategy or other conditioning factors.

## 7. Conclusions

This paper presents implementation of a system that provides data on vehicle usage to establish a more equitable basis for assessment of insurance premiums. A platform on board the vehicle acquires and processes data obtained from the GPS, the EOBD system and a mobile-telephone use detection circuit. A mobile telephone connection transmits these data to a CC, at which the risk the vehicle represents to the insurance company is estimated. This paper’s principal contribution is that it estimates how a vehicle is used and proposes a variety of new solutions (hardware, signal analysis and risk assessment) that will require validation under real-world conditions.

The system proposed is expected to produce economic benefits for both customers and insurance companies, to reduce road traffic and pollution, and, more importantly, to reduce the number of road accidents and their related outcomes. By making minimal modifications to the firmware, other potential applications include image transmission and processing, vehicle location in case of theft (GPS combined with GSM), eCall automated incident detection and reporting (including number of passengers) to the emergency services in the case of an accident, *etc.* The system has particular potential in the fleet management field and could be extended to include other information in addition to vehicle position and speed, such as load data (via RFID) and place and time of loading/unloading, which could be used to prevent theft.

## Figures and Tables

**Table 1.** Results.

	**Area of excessive speed (S_SPEED_) (km^2^/h)**	**Area of square of derivative of speed (S_ACCELERATION_) (km/h^2^)**
Driver 1	3.9000	8.4526e + 04
Driver 2	3.0952	5.2159e + 04
Driver 3	3.7700	4.6422e + 04
Driver 4	20.2868	3.1167e + 05
Driver 5	9.3758	1.0029e + 05
Driver 6	7.4486	1.7478e + 05

**Table 2.** Possible data analysis criteria.

**Criterion**	**Variable**	**Comments**
*How much*	km N_K_	Creation of a penalty system based on number of kilometres (N_K_) travelled by the vehicle within a set time period.
*Where*	Zones T_Z1_, T_Z2_	Definition of various geographic zones, each of which is assigned a coefficient. Vehicle position data are acquired from the GPS. This example considers two zones: T_Z1_, T_Z2_.
*When*	Day/night T_DAY_, T_NIGHT_	Creation of two time bands (day/night), each of which is assigned an appropriate penalty. Chronological data are acquired from the GPS.
*How*	Respect for speed limits S_SPEED_	Estimated as the area between the horizontal line marking the legal speed limit and the extent by which this threshold is exceeded, thereby considering both the amount by which the speed limit is exceeded and the time for which this occurs.
	Aggressive driving S_ACCELERATION_	Analysis of variations in vehicle speed, considering as the variable the area of the vehicle’s acceleration curve.
	Number of vehicle passengers N_OCCUPANTS_	Average number of passengers per N_K_ kilometres travelled. Estimated on the basis of the number of doors opened and the number of seatbelts fastened (data obtained from the EOBD system).
	Mobile telephone use T_MOBILE_	Amount of time the driver uses a mobile telephone whilst the vehicle is in motion.

**Table 3.** Insurance premium calculation.

	**Vehicle A**	**Vehicle B**
N_K_	632	3712
*%T_DAY_, T_NIGHT_*	100.0	80.20
*%T_Zi_*:	100.0	80.20
*N_OCCUPANTS_*:	2	1
T_MOBILE (MINUTES)_	0	57
A_SPEED_	377	2028
S_ACCELERATION_	4.64e + 06	31,100,000
k_i_	k_1_ = 1.00e-05	k_2_ = 40
	k_3_ = 60	k_4_ = 40
	k_5_ = 60	k_6_ = 1.2
	k_7_ = 0.1	k_8_ = 0.001
	k_9_ = 0.000001	
Total premium	€47.20	€130.67
